# Litter type and thickness modulate microclimate and seedling establishment in *Cunninghamia lanceolata*

**DOI:** 10.3389/fpls.2026.1739782

**Published:** 2026-02-26

**Authors:** Bo Liu, Zhengning Wang, Lixin Wang

**Affiliations:** 1 College of Life Sciences, Qufu Normal University, Qufu, Shandong, China; 2 Shandong Key Laboratory of Wetland Ecology and Biodiversity Conservation in the Lower Yellow River, Qufu, Shandong, China; 3 Department of Earth and Environmental Sciences, Indiana University Indianapolis, Indianapolis, IN, United States

**Keywords:** Chinese fir, light interception, litter coverage, microclimate, seedling emergence

## Abstract

Litter cover plays a crucial role in regulating seedling growth by modifying microclimate, yet the joint roles of litter type and thickness remain poorly understood. We used a pot experiment with three litter types (broadleaf *Schima superba*, needle *Cunninghamia lanceolata*, and a mixture), crossed with four thickness levels (0, 200, 400, 800 g·m^-2^) to test how litter modulates microclimate and early performance of *C. lanceolata* seedlings. We monitored soil temperature and soil moisture, photosynthetic photon flux density (PPFD), and the red to far-red ratio (R:Fr) beneath the litter, and measured emergence, survival, morphology, and biomass. Litter cover significantly altered microclimate and in turn affected seedling growth. All litter cover reduced PPFD, with thick *Schima superba* litter nearly eliminating surface light, and R:Fr declined beneath litter. Litter buffered soil temperature, with daytime cooling and nighttime warming, and increased soil moisture, with stronger effects under broadleaf litter than under needle litter. Seedling responses depended on litter type and thickness. Thin to moderate cover enhanced emergence and survival, while thick cover suppressed them, especially under *S. superba* litter. Seedlings displayed shade avoidance, including taller height, reduced root growth, a shift in biomass allocation toward shoots and lower root-to-shoot ratio. Broadleaf litter exerted stronger effects than needle litter, and thickness responses were non-linear, with moderate cover facilitating growth but excessive accumulation inhibiting it. These results clarify the ecological role of litter in regeneration and suggest practical guidelines for forest floor management in subtropical conifer plantations.

## Introduction

Plant litter, as the first physical barrier encountered by seeds after dispersal, plays a crucial role in regulating seed germination and seedling growth (Facelli and Pickett, 1991a; Gavinet et al., 2018; Zhang et al., 2022). This influence is mainly mediated by litter induced changes in microclimate conditions near soil surface (Hovstad and Ohlson, 2008; Veen et al., 2019). Specifically, litter alters key environmental factors such as light availability, soil temperature, and moisture conditions, which subsequently regulate early seedling establishment (Jia et al., 2018). In addition to these physical mechanisms, litter can also exert chemical effects by releasing nutrients and allelopathic compounds that modify soil properties (Ruprecht et al., 2008; Loydi et al., 2015). While the allelopathic effects have been extensively examined, recent studies indicate that physical effects,mediated through microclimate regulation, may exert stronger influences on seedling emergence and early growth (Liu et al., 2017; Chen et al., 2018; Jessen et al., 2023).

The magnitude of these physical effects depends strongly on litter type (Koorem et al., 2011; Wang et al., 2022) and thickness (Jessen et al., 2023; Mingo et al., 2023; Zimmerbeutel et al., 2024). Broadleaf litter generally forms dense and continuous layers that strongly reduce light availability while improving moisture retention and thermal stability near soil surface (Donath and Eckstein, 2008). In contrast, needle litter is usually more porous and translucent, allowing greater light penetration but often providing less effective moisture retention (Sato et al., 2004; Zhao et al., 2019; Li et al., 2020). Increasing litter thickness generally enhances buffering, yet excessive accumulation often suppresses seedling growth by limiting light and forming physical barriers, leading to non-linear responses of seedling growth (Loydi et al., 2013; Zhang et al., 2022; Jessen et al., 2023).

Among the physical effects mediated by litter type and thickness, the regulation of light condition is particularly important (Jia et al., 2018; Hou et al., 2020; Jessen et al., 2023). Litter reduces photosynthetic photon flux density (PPFD) and alters light quality (Schimpf and Danz, 1999), particularly by lowering the red to far-red ratio (R:Fr) (Jankowska-Blaszczuk and Daws, 2007). Such alterations can inhibit the germination of light sensitive seeds and limit photosynthesis (Facelli and Pickett, 1991b). At the same time, litter acts as a thermal and hydrological buffer, moderating soil temperature fluctuations and enhancing moisture retention (Eckstein and Donath, 2005; Li et al., 2014; Xia et al., 2016). This buffering effect is especially important under stressful or variable conditions and can enhance seedling emergence and survival (Hassan et al., 2021).

Although numerous studies have examined the role of litter in shaping microclimate (Ruprecht et al., 2010; Li et al., 2014; Jia et al., 2018), empirical comparisons of the combined effects of litter type and thickness on microclimate and thereby influence seedling growth remains scarce. Many studies treat litter as a homogeneous layer, often overlooking interspecific differences that may lead to contrasting ecological outcomes (but see Ruprecht et al., 2010). Such oversight may obscure important ecological processes, especially in mixed forests and in restoration settings. In these systems, litter composition and thickness can vary greatly and can exert complex influences on seedling growth.

*Cunninghamia lanceolata* (Chinese fir), a conifer of major ecological and economic importance in subtropical China, is widely used in afforestation programs (Yang et al., 2018; Yin et al., 2023). However, extensive monoculture plantations have led to issues such as soil nutrient depletion, biodiversity loss, and regeneration failure. Introducing native broadleaf species such as *Schima superba* into Chinese fir stands has been proposed to improve ecosystem functioning (Liu et al., 2018). *Schima superba* is a dominant evergreen broadleaf tree widely distributed in southern China. It is commonly used in mixed-species restoration programs for Chinese fir plantations because it can improve stand structure, soil quality, and natural regeneration (Yang et al., 2018). Its thick leaves and dense litter structure differ physically from needle litter and can therefore modify near ground microclimate in a distinct way. Such microclimatic changes are expected to translate into differences in seedling establishment in this region. Yet, how variations in litter type (needle vs. broadleaf) and thickness jointly regulate microclimate and, in turn, influence the regeneration success of Chinese fir remains insufficiently understood.

Therefore, this study aimed to elucidate how litter type and thickness modulate microclimate and thereby affect the early growth of *C. lanceolata* seedlings. Specifically, we hypothesized the following:

(1) Litter cover alters the soil microclimate, with greater changes under broadleaf litter than under needle litter.
(2) Owing to its greater capacity for light attenuation and moisture retention, broadleaf litter is predicted to exert stronger effects on seed germination and early seedling growth than needle litter.
(3) The benefits of litter cover for seedling emergence and growth may diminish as litter thickness increases, as the positive effects on soil moisture could be counteracted by the negative impacts on light interception.

## Materials and methods

### Litter and seed collection and pretreatment

Leaves of *C. lanceolata* and *S. superba* and mature seeds of *C. lanceolata* were collected in late November 2021 from a plantation in the Wuyi State-owned Forest Farm (25°02′N, 117°29′E), Zhangping City, Fujian Province, China. The forest farm is located in a subtropical monsoon climate zone characterized by distinct seasons, warm temperatures, and high humidity, with a mean annual temperature of approximately 18 to 20°C. The mean elevation of the area is about 425 m, and soils are predominantly classified as Acrisols (yellow red soil variant). The forest farm has a standing stock volume of 2.722 million m^3^ and a forest coverage of 91.7%. The source stand has been maintained under routine forest farm management, and no additional cultivation treatments were imposed specifically for the purpose of this study.

For litter collection, we selected 20 trees distributed within the plantation stand for each species, and placed one litter trap under the canopy of each tree to collect senesced leaves. Litter collected from all traps was pooled within each species to obtain representative samples. After collection, litter was transported to the laboratory, washed with deionized water to remove dust particles, air-dried at ambient temperature, and stored in paper bags until use.

For seed collection, seeds were collected from at least 10 individual trees and pooled. After collection, seeds were manually cleaned, air-dried, and stored at 4°C until sowing. No formal germination test was conducted prior to sowing; instead, seed quality was ensured through the following screening and pretreatment steps. Before sowing, seeds were sieved to remove very small and very large seeds, and the medium-sized fraction was retained. The retained seeds were then visually screened, and seeds that were malformed, moldy, insect-damaged, or obviously shriveled were excluded. Seeds were subsequently disinfected in 0.3% potassium permanganate (KMnO_4_) solution for 30 minutes, rinsed thoroughly with deionized water, and soaked in water at 45°C for 24 hours. Floating seeds were discarded, and only sinking seeds were used in the experiment.

### Experimental design

The experiment was conducted in a ventilated plastic-film greenhouse at Fujian Agriculture and Forestry University, Fuzhou, Fujian, China (26°04′ N, 119°14′ E). The structure primarily served as a rain shelter to exclude natural precipitation, thereby allowing soil moisture to be regulated via uniform irrigation. Due to open-side ventilation, air temperature closely followed ambient outdoor conditions. Lighting was provided by natural sunlight without supplementation.

Three litter types were used, including *C. lanceolata*, *S. superba*, and a 1:1 (w/w) mixture of both (hereafter referred to mixed). Litter was applied at four coverage levels, 0 g·m^-2^ (control, no cover), 200 g·m^-2^ (thin, ~1.5 cm), 400 g·m^-2^ (moderate, ~3.0 cm), and 800 g·m^-2^ (thick, ~6.0 cm). These litter levels were set within the natural bounds of annual litterfall observed in Chinese fir stands.

The growth substrate consisted of yellow soil, nutrient soil, and river sand (2:1:1, v/v/v). This mixture was filled into opaque plastic pots (19 cm in diameter x 20.5 cm in height). Fifty pretreated seeds were sown evenly in each pot, and the assigned litter treatment was applied uniformly across the soil surface. The control treatment included seven replicates. Each litter type by litter coverage combination also included seven replicates, resulting in 63 pots for litter covered treatments (3 litter types × 3 coverage levels × 7 replicates) and 70 pots in total.

To minimize leaching and disturbance of the litter layer, watering was applied beneath the litter layer. All pots were irrigated with equal volumes of water every three days from May 5 to June 19, and every two days from June 20 to August 6. Pots were randomly arranged and rotated weekly to minimize spatial heterogeneity in light exposure within the greenhouse.

### Environmental monitoring

Light intensity and spectral quality beneath each litter layer were measured using an independent light transmission setup with a quantum sensor (HP350, Hipoint, Taiwan, China) and a red to far-red light sensor (Skye SKR110, Skye Instruments Ltd., UK). To exclude lateral light interference, litter was evenly spread on a transparent glass plate covering an opaque box, with sensor probes positioned centrally beneath the glass. Three replicate setups were established per treatment. Measurements were conducted on three clear days per month from May to August, with readings recorded every two hours from 08:00 to 20:00. The red:far red ratio was calculated as an index of light quality. Soil surface temperature and soil moisture were measured directly in the experimental pots containing seedlings. Soil surface temperature directly beneath the litter layer was monitored using a thermometer (Hengshui Dongtai Instrument and Meter Co., Ltd.) in three replicate pots per treatment during for 7-day monitoring periods each month from May to August. Measurements were taken at fixed times each day (08:00, 11:00, 14:00, 17:00, and 20:00). Soil moisture content was determined gravimetrically by weighed all pots daily at approximately 17:00 from May 5 to August 6.

### Seedling emergence and morphological measurements

Seedling emergence was monitored daily until no new seedlings emerged for 14 consecutive days, marking the end of the germination period. Emergence was defined as cotyledons emerging above the litter layer and becoming exposed to light. After germination, seedlings were cultivated under the same conditions for 60 days. At harvest, five seedlings were randomly selected from each pot for measurements. Root length and total seedling height were measured with a ruler (mm scale), and stem diameter was measured with a digital caliper.

### Biomass and allocation indices

Each seedling was separated into leaves, stems and roots. All samples were oven-dried at 80°C to constant weight, and the dry mass of each component (leaves, stems, roots) was measured separately. To assess seedling growth quality, the stoutness index was calculated as aboveground biomass (leaf + stem mass) divided by seedling height. The following biomass allocation indices were also calculated:


$$AGB=B_{stem}+B_{leaf}$$

$$RSR=\frac{B_{root}}{AGB}$$

$$P:nP=\frac{B_{leaf}}{B_{stem}+B_{root}}$$

In the following equations, *B*_stem_, *B*_leaf_ and *B*_root_ represent stem, leaf, and root biomass, respectively. *AGB* denotes aboveground biomass. RSR denotes the root to shoot ratio. *P:nP* denotes the photosynthetic to non-photosynthetic tissue ratio.

### Data analysis

To evaluate whether density-dependent competition among seedlings influenced the observed treatment effects, we first conducted an analysis of covariance (ANCOVA), with litter thickness as a fixed factor and the number of surviving seedlings per pot as a covariate. The covariate effect was not significant, and inclusion of seedling density did not alter the significance or direction of litter thickness effects. These results indicate that density-dependent competition did not confound the treatment responses; therefore, subsequent analyses were conducted without including seedling density as a covariate.

All data are presented as mean ± standard error. Prior to analysis, the normality of residuals and homogeneity of variances were verified using the Shapiro-Wilk test and Levene’s test, respectively. For each litter type, differences among litter coverage levels were evaluated separately using one-way analysis of variance (one-way ANOVA). When significant effects were detected, *post hoc* comparisons were performed using the least significant difference (LSD) test. Statistical analyses were conducted in SPSS 20.0 (IBM Corp., Armonk, NY, USA), and significance was determined at *p* < 0.05. Figures were produced using Origin 9.1 (OriginLab Corp., Northampton, MA, USA) including line plots, violin plots, and point-and-whisker plots.

## Results

### Effects of litter type and thickness on light interception

**Figure 1:**
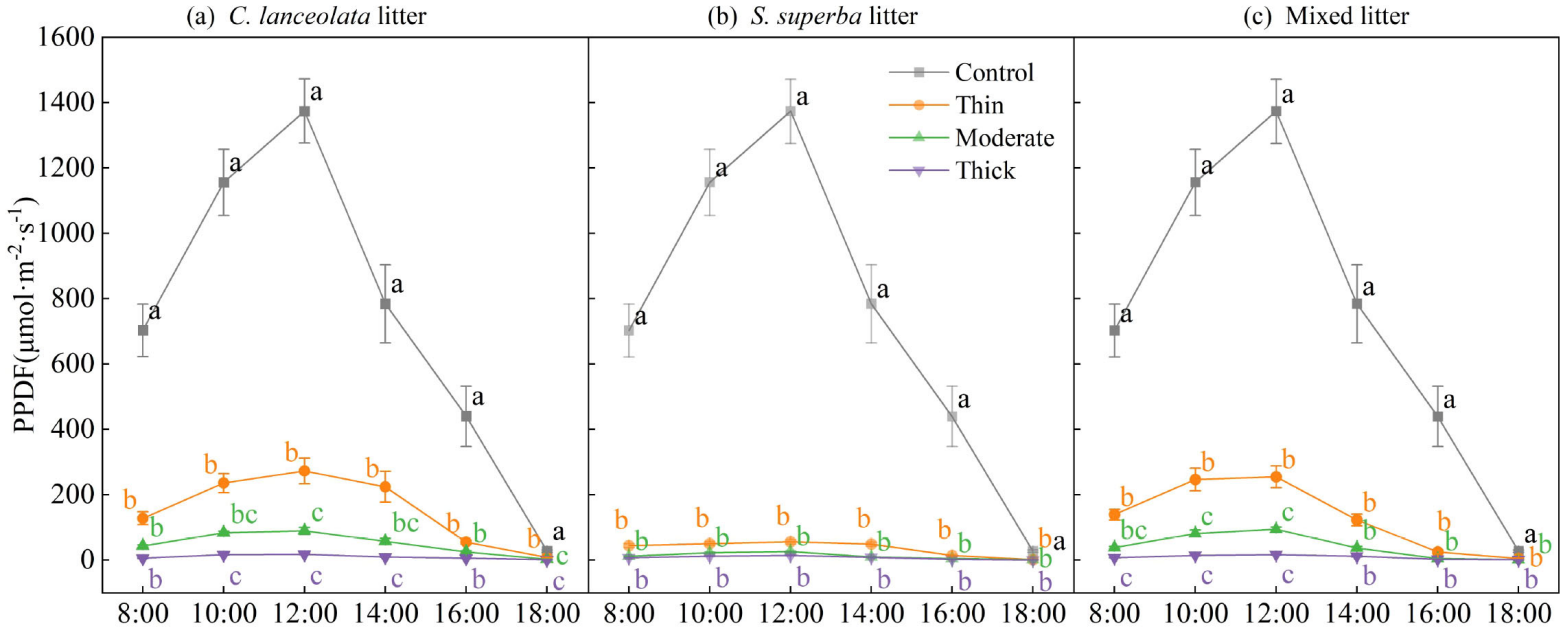
Daily dynamics of photosynthetic photon flux density (PPFD) under litter thickness levels of *C lanceolata***(A)**, *S. superba***(B)**, and mixed litter **(C)**. Values are means ± SE. Different lowercase letters indicate significant differences among litter thickness levels at the same time point (*p* < 0.05).

Photosynthetic photon flux density (PPFD) at the soil surface was strongly influenced by litter type and thickness (**Figure 1**). Across all treatments, PPFD exhibited a typical diurnal pattern, increasing from morning, peaking at noon, and declining in the late afternoon. In the control (no litter), light intensity rose sharply after 08:00, reached a maximum at 12:00–14:00, and then gradually decreased. All litter treatments significantly reduced PPFD relative to the control, and the reduction became more pronounced with increasing litter thickness.

Among litter types, *C. lanceolata* litter (**Figure 1A**) transmitted more light to the soil surface than *S. superba* (**Figure 1B**) and mixed litter (**Figure 1C**). Within each type, thin litter allowed more light penetration than the moderate and thick layers. Thick litter cover, especially in *S. superba* and mixed litter treatments, reduced PPFD to near zero throughout the day.

### Effects of litter type and thickness on the R:Fr

**Figure 2:**
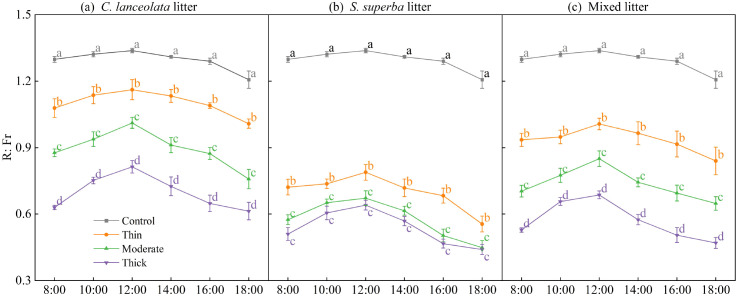
Daily dynamics of the R:Fr under different litter thickness levels of *C lanceolata***(A)**, *S. superba***(B)**, and mixed litter **(C)**. Different lowercase letters indicate significant differences among litter thickness levels at the same time point (*p* < 0.05).

The R:Fr beneath litter cover was significantly influenced by both litter type and thickness, and displayed a typical diurnal pattern (**Figure 2**). Across all treatments, the R:Fr increased slightly around midday (12:00) and declined in the afternoon, but the magnitude of fluctuation was smaller than that observed for PPFD.

The control (no litter cover) consistently maintained the highest R:Fr throughout the day, with significant differences from all litter treatments (P< 0.05). Among the litter types, *C. lanceolata* (**Figure 2A**) generally transmitted higher R:Fr than *S. superba* (**Figure 2B**) and mixed litter (**Figure 2C**).

Within each litter type, thin litter cover resulted in significantly higher R:Fr than moderate and thick covers, while thick litter layers consistently showed the lowest values. This indicates that increased litter thickness enhanced absorption of red light relative to far-red light, thereby reducing the R:Fr.

### Effects of litter type and thickness on soil temperature dynamics

**Figure 3:**
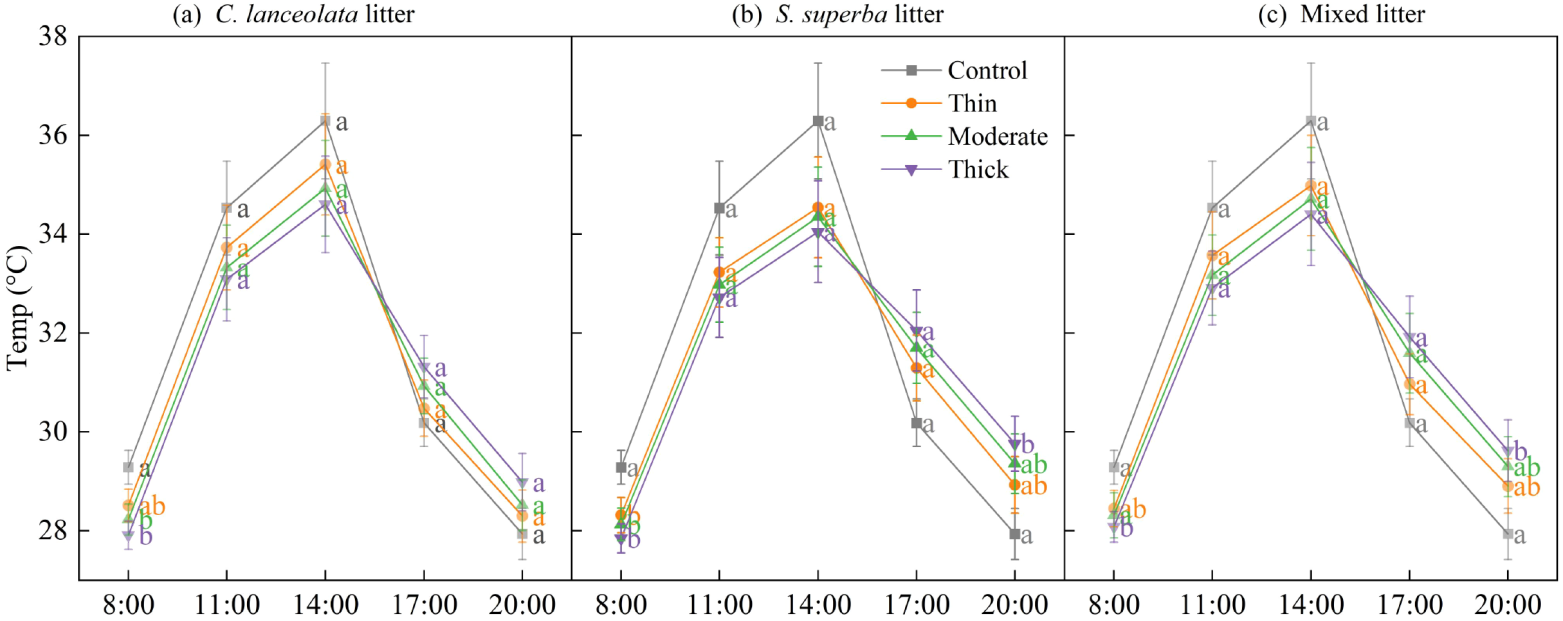
Daily dynamics of soil surface temperature under different litter thickness levels of *C lanceolata***(A)**, *S. superba***(B)** and mixed litter **(C)**. Different lowercase letters indicate significant differences among litter thickness levels at the same time point (*p* < 0.05).

Soil surface temperature showed a distinct diurnal cycle across all treatments, with values increasing from early morning (8:00) to a maximum at 14:00, followed by a steady decline toward 20:00 (**Figure 3**). Compared with the control, all litter treatments significantly reduced soil surface temperature during the warming period (8:00–14:00), indicating a cooling effect of litter cover. In the evening (17:00–20:00), however, plots with litter tended to maintain slightly higher temperatures than the control, reflecting a buffering effect that moderated heat loss, although the differences were not statistically significant at those time points.

Within each litter type, increasing litter thickness generally led to lower daytime temperatures (8:00–14:00) and higher evening temperatures (17:00–20:00), demonstrating a clear buffering effect on soil thermal dynamics. Among the three litter types, *S. superba* litter exhibited the strongest regulation, particularly under the thick treatment, which markedly dampened temperature fluctuations throughout the day.

### Effects of litter type and thickness on soil water content

**Figure 4:**
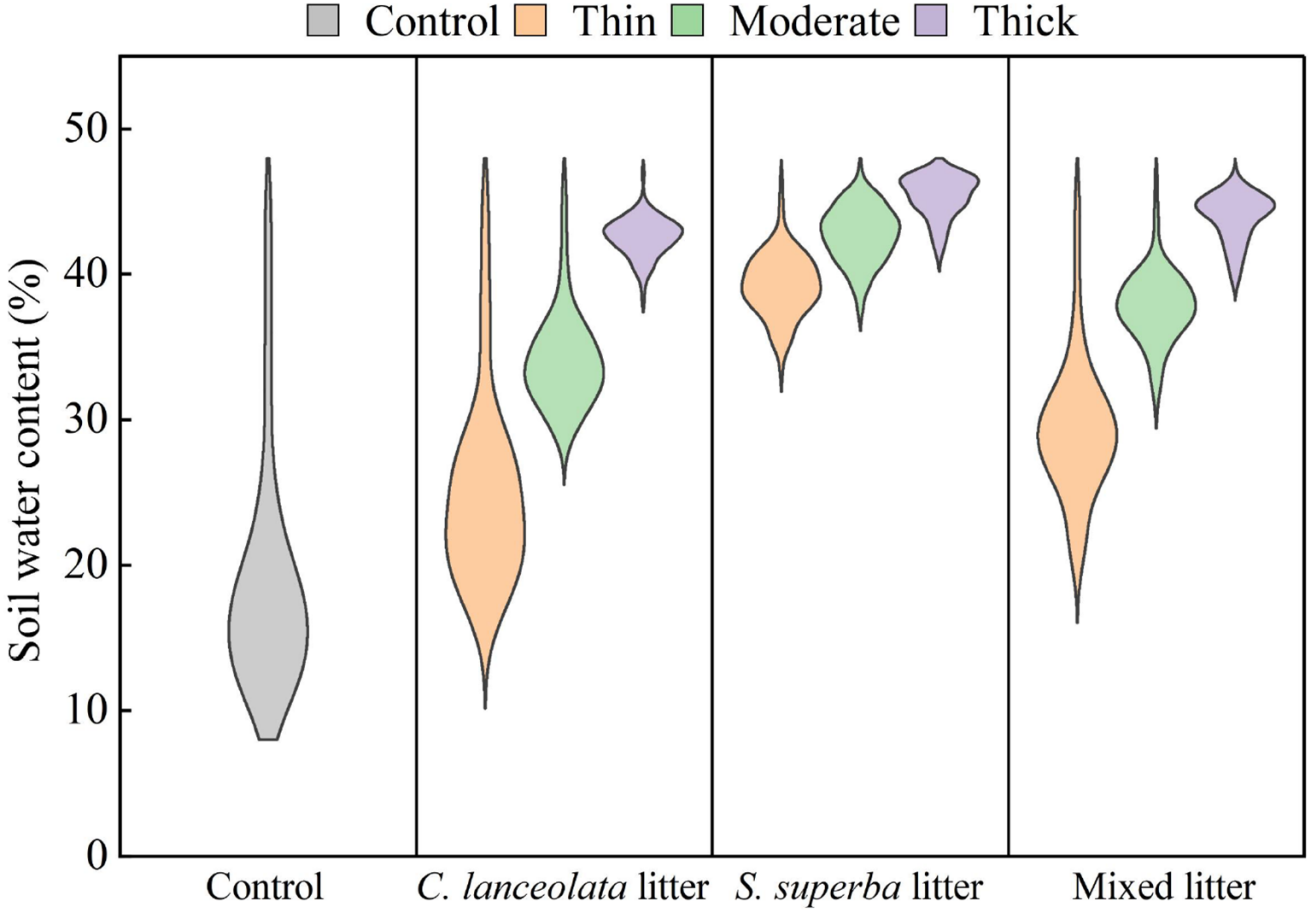
Soil water content (% of soil dry weight) under different litter types and thickness levels. Data were recorded once per day from May 5 to August 6.

Soil water content was significantly influenced by both litter type and thickness (**Figure 4**). Compared with the control, all litter treatments substantially increased soil moisture, highlighting the role of litter in enhancing water retention. Within each litter type, soil water content generally increased with increasing litter thickness, indicating that thicker litter layers were more effective at conserving soil moisture.

Among the three litter types, *S. superba* maintained the highest and most stable soil moisture levels, followed by the mixed litter, whereas *C. lanceolata* showed the weakest effect. In particular, the thin and moderate treatments of *S. superba* sustained relatively high and stable water content, while the thick treatment of *C. lanceolata* was associated with lower soil moisture and greater variability (**Figure 4**).

### Effects of litter type and thickness on seedling emergence and survival

**Figure 5:**
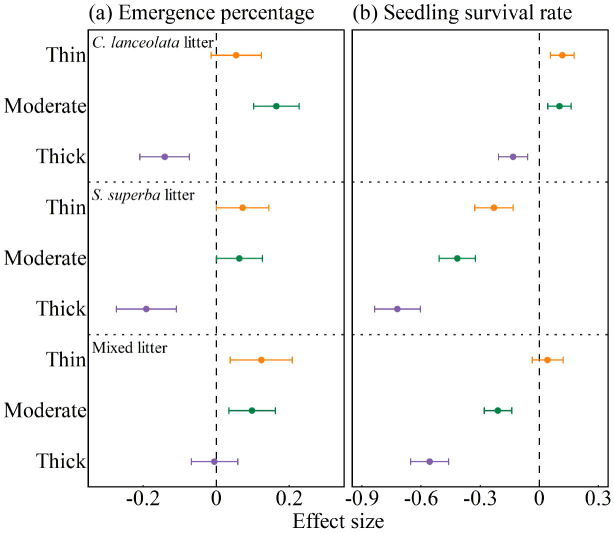
Effects of litter type and thickness on **(A)** seedling emergence, and **(B)** seedling survival under *C lanceolata, S. superba*, and mixed litter covers. Values represent mean effect sizes (relative to the control) with 95% confidence intervals. Positive values indicate positive effects (promotion), whereas negative values indicate negative effects (inhibition) compared to the control. The vertical dashed line denotes zero. Effects are considered significant when the 95% confidence interval does not overlap with zero.

Seedling emergence and survival were strongly influenced by both litter type and thickness (**Figure 5**). Compared to the control, thin and moderate litter layers of all three types showed significant positive effects on seedling emergence, while thick layers exerted significant negative effects. An exception was observed under mixed litter, where thick cover did not exhibit a strong inhibitory effect (**Figure 5A**).

For *C. lanceolata* litter, thin and moderate layers had significant positive effects on survival, whereas thick cover showed a significant negative effect. *S. superba* litter exerted significant negative effects on survival across all thickness levels. Mixed litter showed a weak, non-significant positive effect under thin cover, but significant negative effects under moderate and thick covers (**Figure 5B**).

### Effects of litter type and thickness on seedling morphological traits

**Figure 6:**
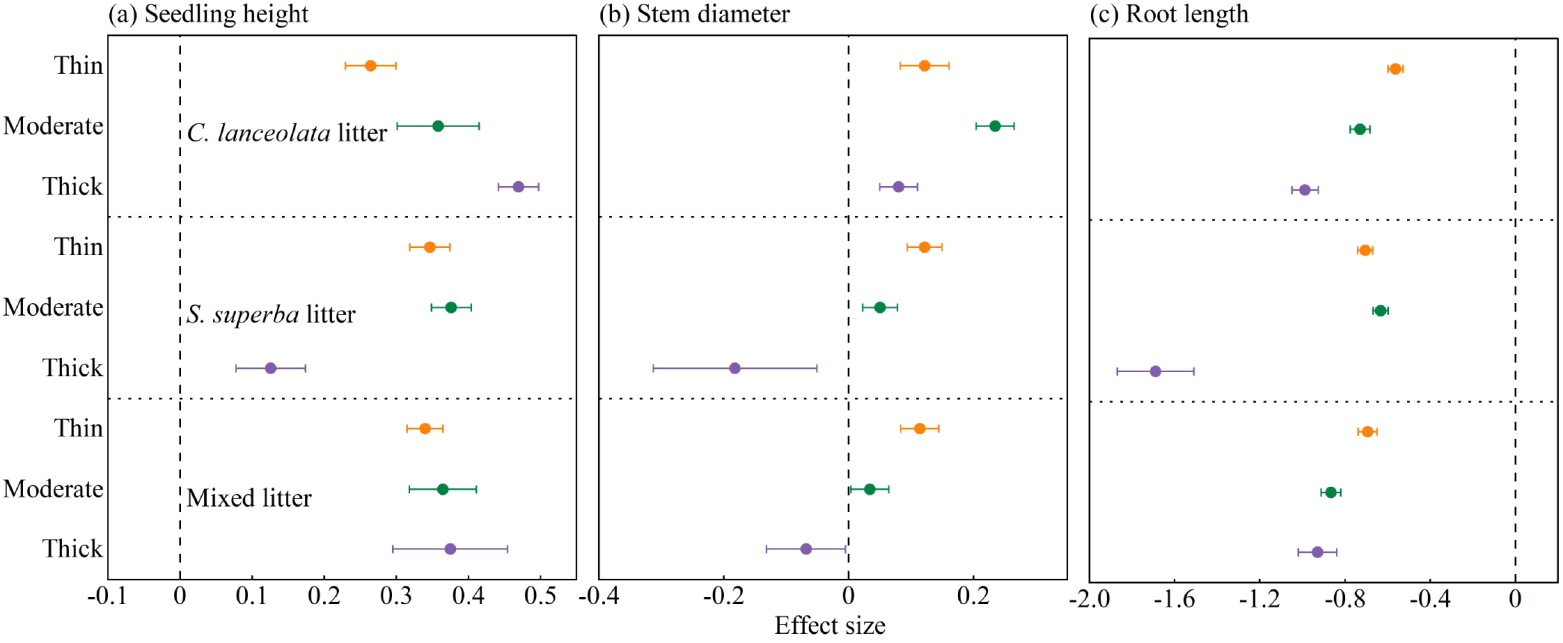
Effect of litter type and thickness on **(A)** seedling height, **(B)** stem diameter, **(C)** root length under *C lanceolata*, *S. superba*, and mixed litter.

Litter type and thickness significantly influenced seedling morphological traits (**Figure 6**). Compared with the control, litter cover at all thickness levels (thin, moderate, and thick) positively affected seedling height across all three litter types (**Figure 6A**). Stem diameter showed significant positive effects under all thicknesses of *C. lanceolata* litter. For *S. superba* and mixed litter, thin and moderate layers had positive effects, but thick layers exerted clear negative effects (**Figure 6B**). In contrast, root length consistently showed negative effects under all litter types and thickness levels (**Figure 6C**).

### Effects of litter type and thickness on seedling biomass and stoutness

**Figure 7:**
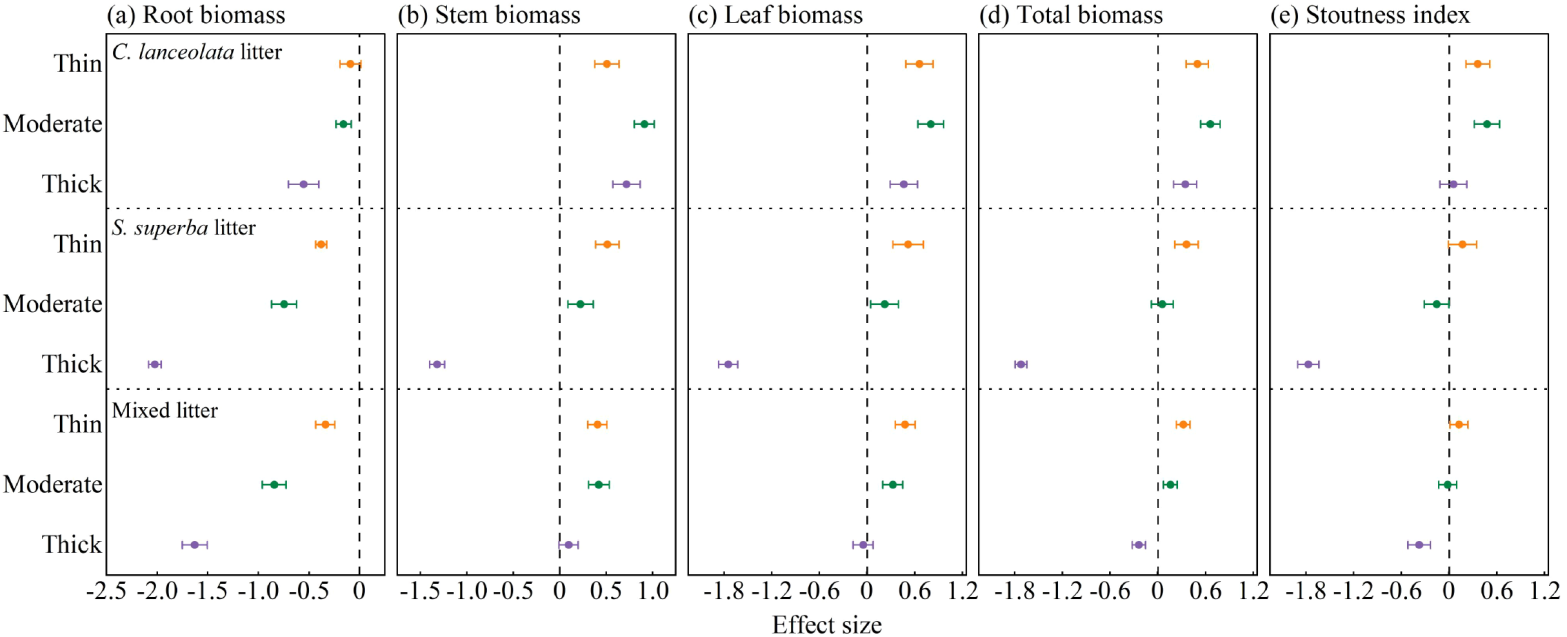
Effects of litter type and thickness on **(A)** root biomass, **(B)** stem biomass, **(C)** leaf biomass, **(D)** total biomass, and **(E)** stoutness index under *C lanceolata*, *S. superba*, and mixed litter.

Litter type and thickness exerted significant effects on seedling biomass accumulation and stoutness (**Figure 7**). Across all litter types, thin, moderate, and thick litter layers consistently imposed negative effects on root biomass (**Figure 7A**). In contrast, the responses of stem, leaf, and total biomass varied with litter type and thickness (**Figures 7B, C**). For *C. lanceolata* litter, all thickness levels produced significant positive effects on stem, leaf, and total biomass. For *S. superba* and mixed litter, thin and moderate layers generally produced positive effects, while thick layers showed significant negative effects on these biomass components. For the stoutness index, *C. lanceolata* litter showed positive effects across all thickness levels (**Figure 7E**). *S. superba* and mixed litter showed positive effects only under thin cover, but shifted to negative effects under moderate and thick layers (**Figure 7E**).

### Effects of litter type and thickness on seedling biomass allocation

**Figure 8:**
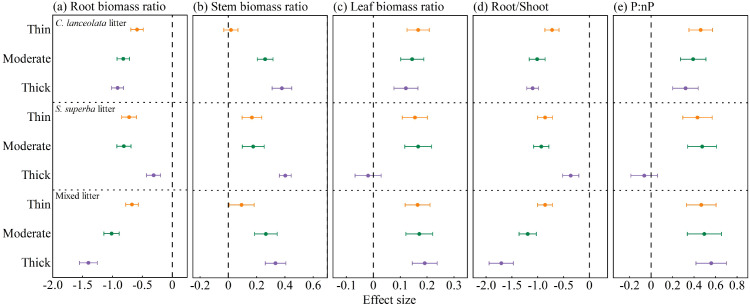
Effects of litter type and thickness on seedling biomass allocation: **(A)** root mass ratio, **(B)** stem mass ratio, **(C)** leaf mass ratio, **(D)** root to shoot ratio (Root/Shoot), and **(E)** photosynthetic and non-photosynthetic biomass ratio (P:nP) unde*r C lanceolata, S. superba*, and mixed litter.

Litter type and thickness significantly influenced biomass allocation patterns of seedlings. Across all treatments, root mass ratio showed consistent negative effects (**Figure 8A**), whereas stem mass ratio was uniformly positively affected (**Figure 8B**). Leaf mass ratio generally exhibited positive responses, with the only exception being a slight negative effect under thick *S. superba* litter (**Figure 8C**).

The root-to-shoot ratio followed a pattern similar to root mass ratio, showing uniformly negative effects across all litter types and thickness levels (**Figure 8D**). By contrast, the P:nP ratio was largely positively affected, particularly under *C. lanceolata* and mixed litter. However, thick *S. superba* litter produced a significant negative effect, reducing the P:nP ratio (**Figure 8E**).

## Discussion

### Influence of litter on microclimate

Our results demonstrated that increasing litter thickness reduced PPFD and the R:Fr beneath the litter. This pattern is consistent with the Beer-Lambert law of light extinction (Facelli and Pickett, 1991a), confirming that litter acts as a light filter, and thicker layers exert a stronger shading effect (Facelli and Pickett, 1991a; Liu et al., 2017).

The extent of light interception varied with litter types. The relatively loose structure of *C. lanceolata* needle litter allowed more PPFD to reach the soil surface than the denser layer formed by *S. superba* broadleaf litter. The strong shading under *S. superba* can be attributed to its broad, dense leaves, which overlap to form compact layers with high light extinction coefficients (Facelli and Pickett, 1991b; Schimpf and Danz, 1999). These structural traits not only restrict direct light penetration but also modify the spectral quality by preferentially absorbing red wavelengths, thereby reducing the R:Fr (Vazquez-Yanes et al., 1990; Facelli and Pickett, 1991b). As the R:Fr modulates phytochrome signaling, these optical changes likely promote shade-avoidance phenotypes in seedlings and inhibit the germination of light-dependent seeds (Vazquez-Yanes et al., 1990; Ruprecht et al., 2010).

In addition to modulating light, litter buffered soil temperature and water availability (Li et al., 2014; Jia et al., 2018). Our study demonstrated that increasing litter thickness markedly reduced diurnal variation in soil temperature and simultaneously improved water conservation, with stronger effects observed under broadleaf than needle litter. During the day, litter layers mitigated excessive surface heating, whereas at night they retained heat and prevented rapid cooling, thereby stabilizing the soil thermal regime. As the litter thickness increased, the amplitude of soil temperature variation decreased due to the insulating effect of the litter, which redistributes heat. These effects were primarily attributable to the low thermal conductivity of organic matter, which inhibits heat exchange between the soil and air (Jia et al., 2018). In addition, litter cover reduced direct solar radiation input and limited convective heat transfer, further contributing to thermal stability (Su and Liu, 2022). By moderating both temperature and moisture dynamics, litter created a more favorable microclimate for seedling emergence and growth (Zheng et al., 2015).

Our results indicated that *S. superba* broadleaf litter modulated microclimate more effectively than *C. lanceolata* needle litter, a difference primarily driven by litter morphology and physical structure. Broadleaf litter forms denser, flatter layers that enhance water retention and shading, leading to more effective buffering of temperature and moisture. In contrast, the looser, more porous structure of needle litter facilitates aeration and drainage but offers poorer insulation and water conservation. These contrasting properties underscore the pivotal role of litter type in shaping microclimate. Our findings are consistent with established theories on the role of litter physical traits in ecosystem functioning (Facelli and Pickett, 1991a) and corroborate recent evidence highlighting interspecific differences in litter-mediated effects (Zimmerbeutel et al., 2024).

### Litter effects on seedling emergence and early growth

Litter cover played a critical role in seedling emergence and early growth by modifying microclimate (Zhang et al., 2022; Jessen et al., 2023). Our results indicated that thin to moderate litter layers promoted seedling emergence and growth, whereas excessive litter cover exerted inhibitory effects (Loydi et al., 2013; Liu et al., 2017; Jessen et al., 2023). These findings were consistent with previous research showing a negative relationship between litter depth and seedling recruitment (Tiebel et al., 2023; Woziwoda et al., 2025). The mechanistic basis for this response lies in the ability of litter to buffer soil moisture and temperature, thereby creating favorable conditions for growth (Xiong and Nilsson, 1999). However, when litter accumulates beyond a threshold, it restricts light penetration and acts as a physical barrier, negatively impacting seedling early growth and survival (Donath and Eckstein, 2008; Liu et al., 2017).

Seedling growth depended on achieving an optimal balance between sufficient moisture and adequate light (Xiong et al., 2003; Jessen et al., 2023; Tiebel et al., 2023). While litter cover can mitigate soil surface desiccation, excessively thick layers markedly limit light availability, suppressing seedling growth (Tiebel et al., 2023). Consequently, although moderate litter coverage facilitated seedling growth, these positive effects tended to diminish or even become detrimental as litter thickness increased (Liu et al., 2017; Zhang et al., 2022). This pattern supports the hypothesis that the benefits of improved moisture retention under thick litter are offset by critically reduced light availability, constraining seedling development.

Litter also shaped seedling morphology in distinctive ways (Koorem et al., 2011; Liu et al., 2017; Zimmerbeutel et al., 2024). In our study, seedlings growing under litter cover exhibited enhanced stem elongation but reduced root biomass, consistent with the shade-avoidance responses reported in previous studies (Li et al., 2014; Asplund et al., 2018; Tiebel et al., 2023). Stem elongation under thick litter represents an adaptive strategy to access available light, albeit at the cost of root development (Liu et al., 2017; Woziwoda et al., 2025). Moreover, seedlings exposed to thick litter exhibited reduced stem diameters, indicating weakened structural stability under low light conditions (Liu et al., 2017). These morphological adjustments result in taller and more slender seedlings with potentially weakened mechanical support, which could undermine survival and stand establishment in field conditions.

Seedlings also exhibited plasticity in biomass partitioning to cope with environmental stressors (Liu et al., 2017; Wang et al., 2022). In our study, litter cover consistently reduced the root-to-shoot ratio while increasing the proportion of biomass allocated to photosynthetic tissues. Seedlings growing under litter tended to invest more resources into above-ground structures, prioritizing stem elongation and leaf expansion to enhance light capture (Ruprecht et al., 2010; Li et al., 2014). While such adjustments improved light acquisition, excessive allocation to shoots at the expense of roots may limit water and nutrient uptake, thereby compromising long-term survival (Woziwoda et al., 2025). The observed decline in seedling stoutness with increasing litter depth further illustrates the trade-offs between enhanced light capture and structural integrity (Liu et al., 2017).

## Conclusions

This study demonstrated that litter type and thickness are critical factors regulating microhabitat and seedling establishment in *C. lanceolata* stands. Broadleaf litter exerted stronger effects on light interception, thermal buffering, and moisture retention than needle litter, and these microclimatic changes translated into stronger impacts on seedling growth. The effects of litter thickness on seedling establishment were non-linear. Thin to moderate litter layers facilitated seedling growth by balancing moisture conservation and sufficient light transmission, whereas excessive accumulation inhibited growth through shading and physical barriers. Seedlings exhibit morphological plasticity to these microhabitat shifts, reallocating biomass toward stem elongation and leaf expansion to enhance light capture under shade. These findings provide mechanistic evidence for the role of litter in shaping regeneration processes and offer practical guidance for silviculture in subtropical conifer plantations. Managing both litter type and depth is essential to optimize microclimate and sustain regeneration, with moderate cover preferred and excessive accumulation avoided.

## Data Availability

The raw data supporting the conclusions of this article will be made available by the authors, without undue reservation.
